# The ancestral chromosomes of *Dromiciops gliroides* (Microbiotheridae), and its bearings on the karyotypic evolution of American marsupials

**DOI:** 10.1186/s13039-016-0270-8

**Published:** 2016-08-03

**Authors:** Elkin Y. Suárez-Villota, Ronie E. Haro, Rodrigo A. Vargas, Milton H. Gallardo

**Affiliations:** Instituto de Ciencias Marinas y Limnológicas, Universidad Austral de Chile, Casilla 567, Valdivia, Chile

**Keywords:** Microbiotheridae, Repetitive DNA, Telomeric sequence, self-Genomic *in situ* hybridization, Constitutive heterochromatin

## Abstract

**Background:**

The low-numbered 14-chromosome karyotype of marsupials has falsified the fusion hypothesis claiming ancestrality from a 22-chromosome karyotype. Since the 14-chromosome condition of the relict *Dromiciops gliroides* is reminecent of ancestrality, its interstitial traces of past putative fusions and heterochromatin banding patterns were studied and added to available marsupials’ cytogenetic data. Fluorescent *in situ* hybridization (FISH) and self-genomic *in situ* hybridization (self-GISH) were used to detect telomeric and repetitive sequences, respectively. These were complemented with C-, fluorescent banding, and centromere immunodetection over mitotic spreads. The presence of interstitial telomeric sequences (ITS) and diploid numbers were reconstructed and mapped onto the marsupial phylogenetic tree.

**Results:**

No interstitial, fluorescent signals, but clearly stained telomeric regions were detected by FISH and self-GISH. Heterochromatin distribution was sparse in the telomeric/subtelomeric regions of large submetacentric chromosomes. Large AT-rich blocks were detected in the long arm of four submetacentrics and CG-rich block in the telomeric regions of all chromosomes. The ancestral reconstructions both ITS presence and diploid numbers suggested that ITS are unrelated to fusion events.

**Conclusion:**

Although the lack of interstitial signals in *D. gliroide*s’ karyotype does not prove absence of past fusions, our data suggests its non-rearranged plesiomorphic condition.

**Electronic supplementary material:**

The online version of this article (doi:10.1186/s13039-016-0270-8) contains supplementary material, which is available to authorized users.

## Background

Chromosome numbers among marsupials ranges from 2n = 10 to 32, with a modal 14-chromosome number (followed by 2n = 22) for the Australian and South American radiation [1–3]. These karyotypes share extensive resemblance in chromosome morphology [4, 5] and G-banding patterns [6], leading to the hypothesis that 2n = 14 is ancestral for marsupials [6, 7]. Thus, larger diploid numbers are assumed to be derived by fissions, as suggested strongly by cytogenetic and phylogenetic comparisons [8–10]. An opposing view on the hybridization patterns of telomeric sequences claimed ancestrality for the 22-chromosome karyotypes and propose subsequent fusions to explain lower numbers [11]. In fact, the 22-chromosome species exhibit only telomeric signals whereas additional centromeric and interstitial telomeric sequences (ITS) suggesting fusion events were detected in 14- and 18-chromosome species [12, 13]. Nevertheless, the colocalization of ITS in heterochromatic pericentromeric regions has been considered to be part of the satellite DNA rather than true telomeric sequences [14–16]. Consequently, interstitial signals outside pericentromeric regions have turned karyotypic ancestrality of American marsupials into an open question [17]. Based on the early divergence of the didelphid *Glironia ventusa* (2n = 18) and by ensuing at least four centric fission/fusion events, a bidirectional trend of karyotypic evolution has been proposed [18, 19]. Nevertheless, the phylogenetic information demands an explanation for the convergence to 2n = 18 in the *Monodelphis* clade [19].

Recent comparative metatherian and eutherian genome assemblies have falsified the fusion hypothesis, thus supporting the ancestrality of the 14-chromosome karyotype [20]. This putative ancestral karyotype is shared by microbiotherians, caenolestids, peramelemorphians, vombatids, and pygmy possums [10]. It includes six large, six medium-sized, and two small sex chromosomes, as inferred from extensive G-banding studies [2, 4, 6] including *Dromiciops gliroides* (2n = 14) [21].

The Microbiotheria is one of the three orders of American marsupials, comprising 12 extinct species and the sole surviving, *D. gliroides* [22, 23]. To inquire whether traces of past fusions could still be detected in *D. gliroides*, fluorescent *in situ* hybridization (FISH) using telomeric probes on mitotic plates were assayed. Since major structural chromosomal rearrangements are associated with cytogenetically detectable heterochromatic regions and repetitive sequences [24–27], we explored both issues by C-banding and self-genomic *in situ* hybridization (self-GISH) [28, 29]. This heterochromatic characterization was complemented with AT and CG-rich banding procedures and centromere identification. To further explore the relationships between fusion events and ITS, their ancestral presence using the phylogenetic tree of Mitchell et al. [30] was reconstructed.

## Material and methods

### Chromosomes

Mitotic plates of two males and one female *D. gliroides* collected in San Martín experimental station of Universidad Austral de Chile (39°38′S, 73°07′W) were used in this study. Chromosomal material was obtained from primary fibroblast cultures derived from ear tissue stored at −196 °C following Verma and Babu [31]. Metaphase spreads for immunofluorescense were prepared according to Zakharova et al. [32]. The ear cell material was collected and cultured previously to this work according to the protocol of the Animal Experimentation Ethics Committee of the Universidad Austral de Chile (UACH) No. 11/09. Cells cultured were cryopreserved for six years in mammal tissue collection of the UACH from where it was thawed and recovered [31].

### FISH, Self-GISH, and immunofluorescence

Telomeric sequence detection by FISH on metaphase chromosomes was performed with the universal telomeric probe (TTAGGG)_n_, generated by PCR and labeled with fluorescein 12-dUTP (Roche Applied Science) [33, 34]. Three different posthybridization washed times (5, 2, and 1 min) with formamide 50 % were used to increase the sensitivity for telomere detection. Unspecific repetitive sequences were detected by self-GISH through hybridization of total genomic DNA probes of *D. gliroides* over its own mitotic plates [29]. Both FISH and self-GISH chromosomes were counterstained with DAPI (4′, 6-diamino-2-phenylindole) and mounted with Vectashield antifade. Mitotic plates were digitally captured at 100x with adequate filters using an Axiolab epifluorescence microscope (Carl Zeiss) equipped with an Axiocam camera.

Centromeres were detected with anti-centromere of polyclonal human antibody (ACA; Cat. No. 15–235, Antibodies Incorporated). Goat anti-human IgG conjugated with Texas Red (Cat. No.23773-2, Bioscience) was used as secondary antibody. Both antibodies were diluted in 1:100 PBS and applied on mitotic plates [32]. Mitotic plates were mounted and captured as described previously.

### Chromosome banding

C-banding was conducted using the Ba(OH)_2_ treatment at 46 °C for 3–4 min [35]. Given that AT and CG staining denote chromatin’s nucleotide composition, blocks of AT-rich sequences were detected with methyl-green/DAPI [36] whereas CG-rich regions were identified through chromomycin [37]. C-bands were observed under the microscope with a halogen lamp whereas a mercury lamp with adequate filters was used for chromomycin and methyl-green/DAPI staining. Banding images were captured as described above.

### Ancestral state reconstruction

To inquire onto the ancestrality of diploid numbers and ITS presence in American marsupials, Bayesian trait reconstruction onto the phylogeny of Mitchel et al. [30] was performed using BayesTraits v2.0 [38]. This phylogeny includes the largest number of characters and marsupial species. The MCMC multistate module was implemented using a compilation of cytogenetic data listed in Additional file 1: Table S1. The analysis was run for 10^6^ iterations, sampling every 10^3^. The stationary phase was checked using Tracer version 1.6 [39] and sample points prior to the plateau phase were discarded as burn-in. To test significance, Bayes Factor (BF), was estimated as the difference in the log’s marginal likelihood between the data and the theoretical model containing restricted transition rates. Marginal likelihoods were estimated using stepping stone sampler [38, 40].

## Results

### FISH, Self-GISH, and immunofluorescence

**Fig. 1:**
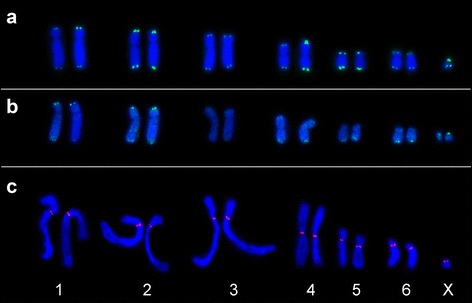
Molecular cytogenetic analyses on *D. gliroides* chromosomes. **a** FISH over a male mitotic plate, using the telomeric probe. Note the absence of interstitial signals in all chromosomes. **b** Self-GISH on a female mitotic plate. Note the strong fluorescent signals in some telomeric regions and a dot-like fluorescent pattern in all chromosomes, except pair 3. **c** Immunoassay over male mitotic plates using the anti-centromere of polyclonal human antibody. Colocalization of red fluorescent signals with the primary constriction is observed

Telomeric, but no centromeric or interstitial signals were detected on all chromosomes of *D. gliroides* (Fig. 1a)*,* regardless of stringency conditions (Additional file 2: Figure S1).

Intensive signals of high repetitive sequences in telomeric/subtelomeric regions together with dot-like fluorescent patterns in all chromosomes (except in pair 3) were detected by Self-GISH. Signals were also observed in the short arms of X-chromosomes and on the long arm of chromosomes 5 and 6 (Fig. 1b).

**Fig. 2:**
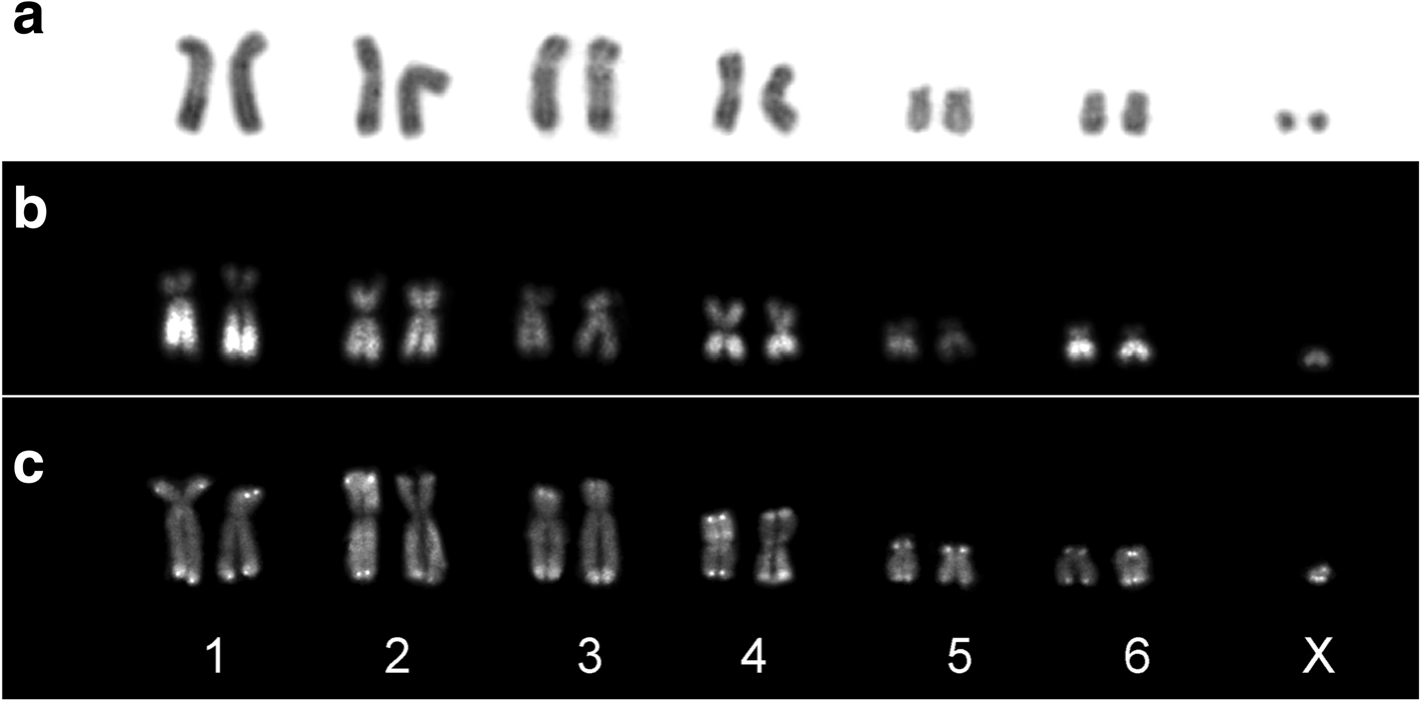
Banding patterns of *Dromiciops gliroides*. **a** C-banding of a female mitotic plate. Note the sparse distribution pattern and absence of pericentromeric heterochromatin. **b** CG- fluorescent banding of a male mitotic plate. Note the intensively stained blocks of CG-rich sequences in telomeric/subtelomeric regions. **c** AT- fluorescent banding of a male mitotic plate. Note that AT-rich sequences are the inverse of the CG- banding pattern

As expected, immunoassays allowed the detection of *D. gliroides*’ centromeres in primary constrictions devoid of heterochromatin (compare Figs. 1c and 2a).

### Chromosome banding

No centromeric/pericentromeric, but sparse distribution of heterochromatin along all chromosomes resulted from the C-banding procedure (Fig. 2a). Nevertheless, weak heterochromatic areas were observed in telomeric/subtelomeric regions of chromosomes 1–3, and in both arms of chromosome 4 (Fig. 2a).

Large AT-rich blocks were detected in the interstitial regions of chromosomes 1, 2, 4, and 6. Stronger signals were observed in the long arm of chromosomes 1 and 2, and in both arms of chromosomes 3 and 4 (Fig. 2b). GC signals were restricted to telomeric/subtelomeric regions of all chromosomes (Fig. 2c). As reported previously, the Y chromosome was not detected in any of the 150 metaphase plates of each adult male analysed [41].

### Ancestral state reconstruction

**Fig. 3:**
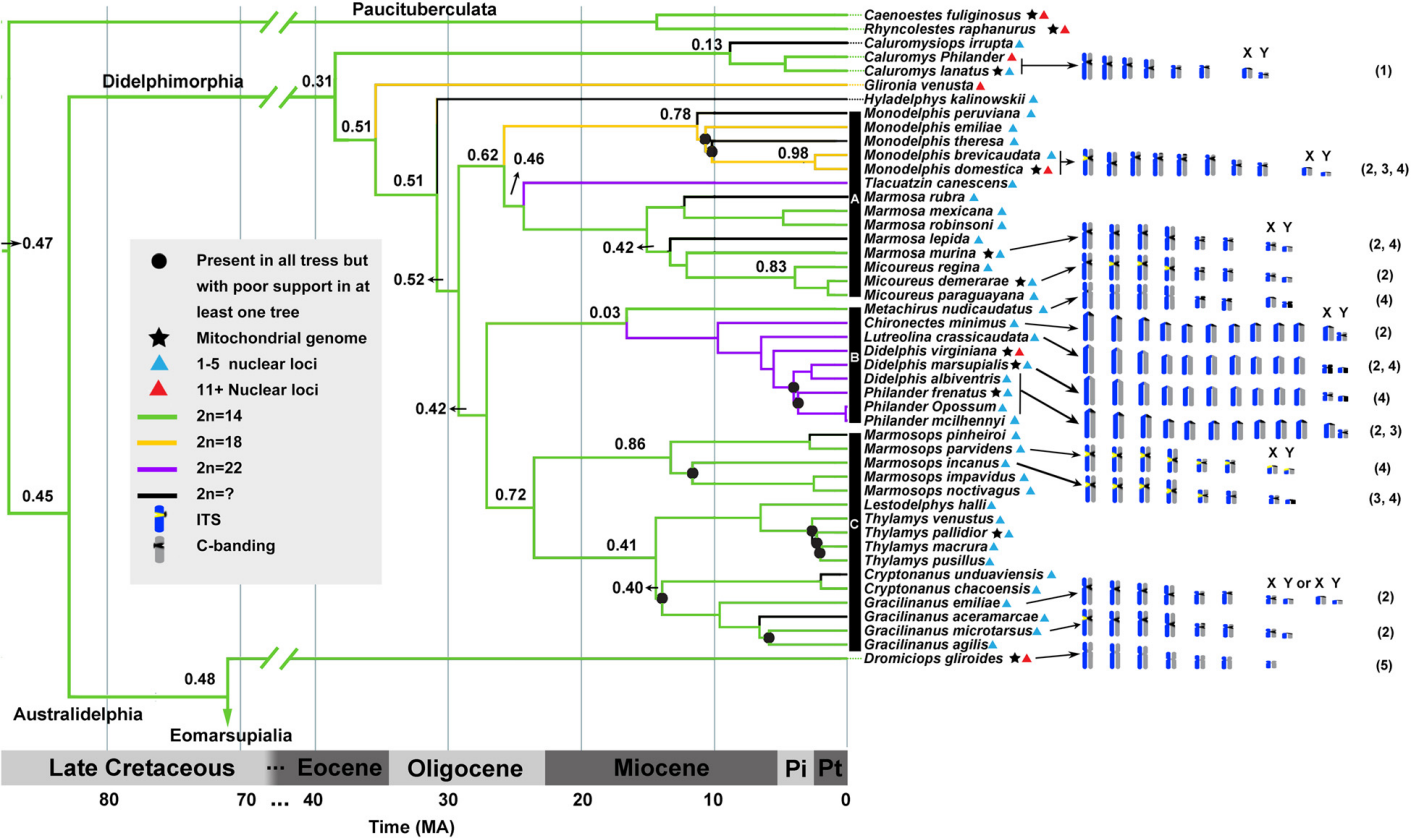
Phylogenetic tree of American marsupials [30] depicting ancestral diploid number and ITS presence reconstruction. Branch colors indicate diploid number reconstructed with BayesTraits. Numbers above branches represent probability for each ancestor having ITSs. Cytogenetic data depicted at right follows: 1 = Sousa et al. [56]; 2 = Carvalho and Mattevi [12]; 3 = Svartman and Vianna-Morgante [13]; 4 = Pagnozzi et al. [15]; 5 = this study. Additional information is listed and coded in Additional file 1: Table S1

Both ITS presence and ancestral chromosome number for the American marsupials are shown in Fig. 3. Thus, 2n = 14 probably represents the ancestral diploid number of all marsupials including Paucituberculata, Didelphimorphia, and Australidelphia (Fig. 3, green branches). The transition from 14 to 22 chromosomes occurs in clades A and B, at about ∼ 24 and ∼ 18 Mya, respectively (Fig. 3, purple branches). The transition from 14 to 18 chromosomes would occur about ∼ 36 Mya in the *Glironia venusta*’s lineage and about ∼ 26 Mya in the *Monodelphis* clade (Fig. 3, orange branches). Considering the distribution of telomeric sequences in only 17 American species, low confidence (log BF <2) was obtained by ITS reconstruction. Nevertheless, the probability for each ancestor having ITSs is given, such that their highest probabilities would indicate ITS’ ancestral presence (Fig. 3, derived lineages within A and C clades).

## Discussion

Sparse heterochromatin distribution and few dispersed repetitive sequences on interstitial and centromeric regions characterize the karyotype of *D. gliroides* (Figs. 1b and 2a). This features are coherent with DNA annealing data depicting few repetitive DNA and higher effective concentration of single-copy sequences in *D. gliroides* relative to other marsupials [42]. The sparse labeling pattern detected by self-GISH along some chromosomes suggests a correspondence with specific genomic repetitive sequences (e.g. interspersed transposons and dispersed repetitive sequences; Fig. 1b). In fact, a dot-like fluorescent pattern is associated with the enrichment of retroelements on some chromosomes of the rodent *Octomys mimax* [43], *Chionomys nivalis* [44], and several *Microtus* species [45]. The lack of both repetitive sequences and pericentromeric heterochromatin has been associated with centromeric shifts and neocentromerization in cattle and in the marsupial genus *Petrogale* [46, 47]. This pattern is intriguing considering its role as substrates and as possible stabilizer for centromere formation and function [48]. Further satellite DNA studies, the analyses of centromere DNA-binding proteins, and the epigenetic studies of centromeric chromatin will shed light on the factors that have shaped this peculiar heterochromatic pattern.

GC-rich telomeric sequences detected in all *D. gliroides*’ chromosomes colocalize with most repetitive sequences detected by FISH and self-GISH (Figs. 1a, b and 2c). Since neither centromeric nor interstitial signals were detected by any technique applied, the lack of ITS is strongly suggested (Figs. 1a, b and 2c). It could be argued that this might be due to either loss or chromatin modification after fusion, as reported in *Mus* [25]. Assuming fusions, it seems unlikely that all ITS expected in *D. gliroides* were lost. Thus we hypothesize that ITS have never existed in *D. gliroides* chromosomes and support the notion that its karyotype does not fit into the fusion model. The undetection of ITS fits well with the cytogenetic/phylogenetic inference and genome assemblage comparison [10, 20]. This hypothesis is further supported by the lack of interstitial C-bands (Fig. 2a), as the opposite is predicted if fusions took place [25–27]. Our results agree with the cytogenetic studies on Australian genera *Macropus*, *Petrogale,* and *Thylogale* [16, 46, 49] that support the ancestrality of the 14-chromosome karyotype. Chromosome painting onto the chromosomes of *D. gliroides* will clarify its ancestral 14-chromosome condition by showing conserved regions and possible inversions.

Previous and varied phylogenetic studies have indicated that *D. gliroides* is more related to the Australasian than to the South American radiation [21, 30, 42, 50, 51]. This is consistent with the microbiotherid’s oldest fossil known from the Early Paleocene, when Australia, South America and Antarctica were still connected [52]. These two facts support *D. gliroides* as a living fossil that links the American and the Australian radiations. Consequently, its cytogenetic features might well be supporting data for the marsupial ancestral state reconstruction. In fact, the shared 14-chromosome karyotypes of *Dromiciops*, *Caenolestes*, *Rhyncholestes,* and *Caluromys* allow us to confidently (>90 %) support their ancestrality, also extended to the Didelphidae and Caenolestidae, as previously advanced [10, 20].

The lack of ITS in *Dromiciops* and *Caluromys* makes improbable their existence in the basal nodes of marsupial phylogeny (Fig. 3). Assuming their absence (*P* < 0.5), its onset would have been after the divergence of *Glironia venusta* from the remaining didelphids, more recent than ∼ 36 Mya (Fig. 3). Given the small probability for ITS presence, the transition from 14 to 18 chromosomes would be unrelated to fusion events (Fig. 3, orange branches). In the same line, the onset of marsupial ITS would be traced after the dispersion from South America to Australia, during the Paleocene [53, 54], falsifying the predictions derived from the fusion hypothesis [11–13].

The 14-chromosome ancestors of *Gracilanus* and *Marmosops* have a high probability of having ITS while the 14-chromosome ancestor between them and *Metachirus* has a low corresponding probability (Fig. 3). A similar situation is observed in the ancestor of the *Micoureus* clade with respect to *Marmosa murina*. Both instances illustrate the conservation of 14 chromosomes, indicating that ITS acquisition was unrelated to the fusion events. ITS presence might well represent signals of satellite DNA associated to constitutive heterochromatin, as advanced [14–16, 55]. Since ITS presence colocalizes with the constitutive heterochromatin, and its presence occurs in derived 14- and 18-chromosome species depicted in the phylogenetic tree, the fusion hypothesis rendered highly improbable.

## Conclusions

Neither centromeric nor interstitial signals reflecting fusions were detected in the “living fossil” *D. gliroides* by either classical or molecular cytogenetics. The ancestral reconstruction of diploid number as well as the ITS absence suggested their decoupling from past fusion events. Therefore, our data give additional support to the notion that fission events from an ancestral 14-chromosome condition explain the high chromosome numbers in the karyotypic radiation of American marsupials.

## Abbreviations

BF, Bayes factor; DAPI, 4′, 6-diamino-2-phenylindole; dUTP, deoxyuridine Triphosphate; FISH, fluorescent *in situ* hybridization; IgG, immunoglobulin G; ITS, interstitial telomeric sequences; MCMC, Markov chain Monte Carlo; min, minute; Mya, million years ago; PBS, phosphate buffer solution; self-GISH, self-genomic *in situ* hybridization; UACH, Universidad Austral de Chile

## Additional files

Additional file 1: Table S1. Cytogenetic data used for the reconstructions of ancestral diploid numbers and presence of interstitial telomeric signals (ITS). Multi states codes used in BayesTraits were A: 2n = 14, B: 2n = 18, C: 2n = 22, D: 2n = 10, E: 2n = 12, F: 2n = 16, G: 2n = 18, H: 2n = 20, I: 2n = 24, J: 2n = 32; K: presence of ITS, L: absence of ITS. Missing data code (−) was used for diploid numbers and/or ITS of species without these cytogenetic data. (XLS 44 kb) Additional file 2: Figure S1. Fluorescent *in situ* hybridization over *D. gliroides* mitotic plates using the telomeric probe. Posthybridization washes with formamide 50 % for a: 5 min, b: 2 min, and c: 1 min were tested. Note the absence of interstitial signals in all chromosomes. (JPG 691 kb)
