# Supplementary figures and images for: The ancestral chromosomes of Dromiciops gliroides (Microbiotheridae), and its bearings on the karyotypic evolution of American marsupials
Source: Mol Cytogenet. 2016 Aug 3;9:59. doi: 10.1186/s13039-016-0270-8 (PMC4971695; doi:10.1186/s13039-016-0270-8)

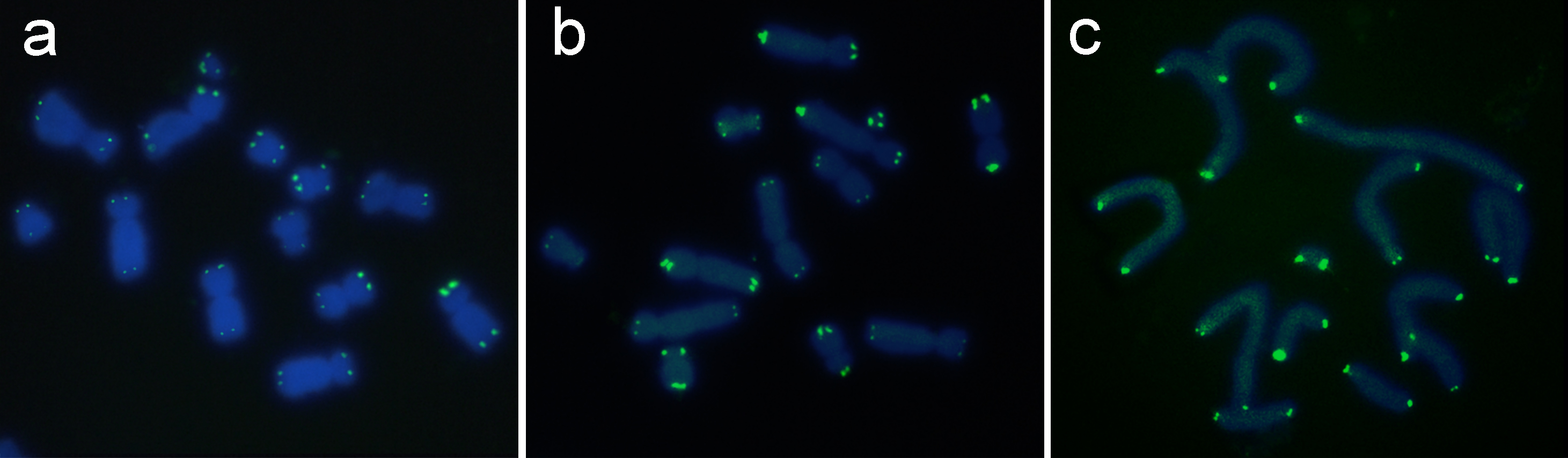

Supplement: Additional file 2: Figure S1. — Fluorescent in situ hybridization over D. gliroides mitotic plates using the telomeric probe. Posthybridization washes with formamide 50 % for a: 5 min, b: 2 min, and c: 1 min were tested. Note the absence of interstitial signals in all chromosomes. (JPG 691 kb) [file 13039_2016_270_MOESM2_ESM.jpg]
